# Soft Robotics in Upper Limb Neurorehabilitation and Assistance: Current Clinical Evidence and Recommendations

**DOI:** 10.1089/soro.2024.0034

**Published:** 2025-06-11

**Authors:** Natalie Tanczak, Aaron Yurkewich, Francesco Missiroli, Seng Kwee Wee, Simone Kager, Hyungmin Choi, Kyu-Jin Cho, Hong Kai Yap, Cristina Piazza, Lorenzo Masia, Olivier Lambercy

**Affiliations:** ^1^ Singapore-ETH Centre, Future Health Technologies Programme, Singapore, Singapore.; ^2^ Department of Health Sciences and Technology, Rehabilitation Engineering Laboratory, ETH Zurich, Zurich, Switzerland.; ^3^ Mechatronics Engineering, Ontario Tech University, Oshawa, Canada.; ^4^ Institute of Computer Engineering (ZITI), Faculty of Engineering Sciences, Heidelberg University, Heidelberg, Germany.; ^5^ Clinic for Advanced Rehabilitation Therapeutics (CART), Tan Tock Seng Hospital, Singapore, Singapore.; ^6^ Institute of Rehabilitation Excellence (IRex), Tan Tock Seng Hospital, Singapore, Singapore.; ^7^ Singapore Institute of Technology, Singapore, Singapore.; ^8^ Department of Mechanical Engineering, Soft Robotics Research Centre (SRRC), Seoul National University, Seoul, Republic of Korea.; ^9^ Roceso Technologies, Singapore, Singapore.; ^10^ School of Computation, Information and Technology and Munich Institute of Robotics and Machine Intelligence (MIRMI), Technical University of Munich, Munich, Germany.

**Keywords:** soft robotics, neurorehabilitation, wearable, portable, medical devices, clinical evidence, recommendations

## Abstract

Soft robotics is gaining interest in rehabilitation applications, bringing new opportunities to offset the loss of upper limb motor function following neurological, neuromuscular, or traumatic injuries. Unlike conventional rigid robotics, the added softness in linkages or joints promises to make rehabilitation robots compliant, which translates into higher levels of safety, comfort, usability, and portability, opening the door for these rehabilitation technologies to be used in daily life. While several reviews documented the different technical implementations of soft rehabilitation robots, it is essential to discuss the growing clinical evidence on the feasibility and effectiveness of using this technology for rehabilitative and assistive purposes, whether softness brings the expected advantages from the perspective of end users, and how we should proceed in the future of this field. In this perspective article, we present recent clinical evidence on how 13 different upper limb devices were used in both controlled (clinical) and uncontrolled (at home) settings in more than 37 clinical studies. From these findings and our own experience, we derive recommendations for future developers and end users regarding the design, application, and evaluation of soft robotics for upper limb rehabilitation and assistance.

## Introduction

Many neurological, neuromuscular, or traumatic injuries lead to disabling and long-lasting upper limb deficits that have a significant impact on the quality of life and independence of the affected individuals. The potential of robotics to complement conventional upper limb rehabilitation has been widely reported in the literature, as robotic tools can provide intensive and well-controlled training conditions, possibly without requiring additional clinical manpower resources.^1^ Recent years have seen significant developments in wearable robotics, with the additional objective of combining therapy and assistance,^2–5^ where robotics can compensate for upper limb deficits and augment patients’ remaining motor function during daily life tasks. This could help increase rehabilitation dose in an engaging and transferable manner outside of laboratory/clinical settings.

From a technical perspective, many different solutions have emerged based on different design approaches, relying on conventional rigid robotics or, more recently, on soft robotics.^6–8^ Traditional assistive and rehabilitation robots typically adopt rigid underlying structures and components that excel in scenarios demanding precise and controlled movements.^9^ Their structural stability is essential to assist patients with severe mobility limitations, enabling accurate trajectory tracking and reliable force application. While capable of high performance, existing conventional rigid robotics can pose challenges. Their lack of flexibility restricts their ability to conform to both objects and human anatomy. This can lead to limited comfort and potential safety concerns, such as skin abrasion or strains on joints and ligaments.^10^ Furthermore, their complexity and high costs raise the question of their applicability, especially when extending the technology to patient’s homes. Soft robotics, where designs rely on soft deformable linkages or joints to transmit assistive forces to a user, are designed to be inherently compliant, which makes them safer for both the end user and their surrounding environment. This softness can also provide and improve the devices’ comfort, usability, and portability.^8,11^ Particularly in the context of neurorehabilitation and assistance, soft robotics have the added benefit of being able to compensate for loss of motor function without interfering with anatomically correct movements. Due to the materials used to construct soft robots, they can more closely mimic the flexibility and adaptability of biological tissues. As a result, there is a growing agreement that soft robotics could help fill the need and address existing challenges in traditional robotics in this specific context of use. However, how the expected advantages of soft robotics transfer to benefits for the end users of such technologies (e.g., patients and clinicians) remains underevaluated.

While existing reviews on the development of soft robotics for upper limb neurorehabilitation and assistance primarily focus on the description of technical solutions and their implementation,^12^ here in this perspective article, we compile and provide our insights into the growingly available clinical evidence and user experiences from various studies involving patients with upper limb impairment, with the aim of evaluating the promise of soft robotics for this application field. In this work, we build on the experience and opinions of the authors and report on the discussions initiated during a workshop at the IEEE International Conference on Soft Robotics (RoboSoft 2023). The objectives of this perspective article are to (1) provide a brief overview of key current and future applications of soft robotics in neurorehabilitation and assistance, (2) review and discuss recent clinical evidence, and (3) highlight the implementation challenges and opportunities for the development, evaluation, and acceptance of such technologies. Based on this, we provide recommendations for future developments, applications, and clinical validations of soft robotics in upper limb neurorehabilitation and assistance.

## Technical and Clinical Motivation: Why Do We Need Soft Rehabilitation Robots?

Recently, the field of rehabilitation robotics has experienced a shift where soft robots complement rigid ones. These soft devices expand the range of the target population, now allowing personalizable assistance for more mildly impaired patients. Traditional rigid devices are typically constructed from harder materials, which provide them with robustness and durability, which are required for exact and highly repetitive movements in neuromuscular training where higher forces are involved. This, however, often makes the devices bulky and heavy, thus limiting their usage to clinical settings and making them suboptimal for mild impairments or long-term daily use. In contrast, soft exoskeletons are lightweight and flexible and conform naturally to human movement, providing a more personalized and comfortable fit. By being less obtrusive and more adaptable, the devices can offer continual gentle assistance to mildly or moderately impaired individuals regardless of the environment. Ultimately, this fundamental rethinking of how robotic technologies are designed should focus on more compact, more robust, and simpler (e.g., to operate, set up, and adapt to a user) solutions. We would argue that the features of soft robotics are instrumental in covering the whole spectrum of a therapeutic approach, from rehabilitation to assistance.

Commonly reported obstacles of conventional, rigid rehabilitation devices are their complexity and limited usability, often requiring skilled personnel for operation, which ultimately hamper the acceptance and utilization of rehabilitation technologies. However, simply reducing complexity typically compromises the functionality of the technology, for example, decreasing the number of mechanical parts or joints to be actuated and controlled. Taking inspiration from nature, it has been proposed to incorporate soft elastic components into the rigid physical structure of mechanical systems to facilitate natural motions that align with human motor control principles. For example, leveraging on the well-described motor synergies^13^ allows for the design of assistive hand prostheses or orthoses, such as the SoftHand Pro (SHP), that can support the most common grasping patterns involving all finger joints with only one or two actuated degrees of freedom.^14–17^ The SHP, with its compliant design and adaptability, provides high robustness and can accomplish complex physical interaction tasks while demonstrating good grasp capabilities, as was experimentally validated in real-world applications during CYBATHLON 2016 and 2020.^16,18^ Such a bioinspired approach allows, via softness in the robotic fingers, to decrease both the mechanical complexity and the load on the user to control/trigger assistance from such devices. In addition, soft systems can distribute pressure induced during force transmission over larger areas and can conform to the body’s internal structure to enhance comfort. Another advantage of soft robotics lies in their intrinsic compliance and adaptability. For example, in the case of exoskeletons, this allows for partially adjusting for joint misalignment, a key issue in wearable robotics.^19^ In that sense, soft robots can help simplify donning and doffing procedures for patients and clinicians, an essential step toward higher acceptance. By creating more natural movement patterns, soft systems also establish safer interactions between the device and user, a crucial aspect in clinical contexts characterized by delicate and variable motions.^20,21^ The inherent compliance of soft robots minimizes the risk of unintended injuries, notably enhancing patient safety during rehabilitation. However, this does not eliminate the importance of good design. Designers should review and iterate through designs to optimize the construction to ensure safety, comfort, and suitable force transmission.

**FIG. 1.**
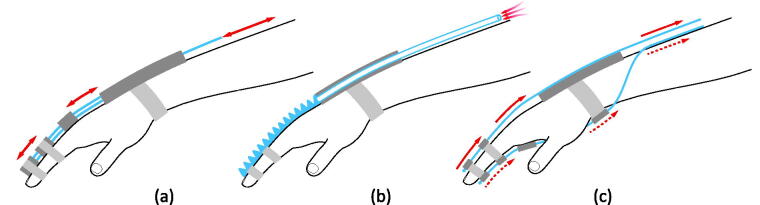
Examples of different possible soft robotic hand exoskeleton designs: **(a**) spring blade, **(b)** pneumatic, and **(c)** cable driven. The soft components are represented in *blue*, with actuation methods depicted in *red*.

Softness in rehabilitation robotics can be found in various technical forms, typically grouped by their actuation methods (Fig. 1). Many existing reviews^8,12,22–25^ already summarized the current state of the art of this technology. Pneumatics are the most common soft, wearable upper limb robotics, accounting for more than half of devices in literature.^8^ They rely on compressed air to inflate elastomer chambers, which, depending on their geometries, can bend, extend, contract, or even elongate. While it is the most popular option, the required air tanks could influence the portability of these devices. As another approach to implement softness, cable-driven systems transmit forces by applying tension on strategically placed anchor points embedded within textiles to promote movement. These designs can be more compact due to the small form factor of the winding electric motors; however, cable transmission comes with the challenges of friction, cable loosening, and backlash. Spring blades were also proposed to transmit forces in hand exoskeletons by transferring linear motion inputs into a rotational movement, thus flexing and extending the fingers. Finally, hybrid blends of the aforementioned categories can be combined, taking advantage of the strengths of each respective actuation strategy.

Soft rehabilitation robots are designed to be lightweight, compliant, and flexible, resulting in safe human interaction.^26^ From a clinical perspective, these features make them attractive for functional assistance in rehabilitating neurological patients, specifically those with muscular weakness, flaccid upper limbs, and minimal spasticity (Modified Ashworth Scale ≤2). Due to the inherent safety and compliance in their design, the technology can be used for early commencement of upper limb rehabilitation, which has been shown to lead to better functional outcomes.^27,28^ In addition, compliance (e.g., during grasping assistance) may be ideal to support functional tasks involving manipulating real objects as part of therapy sessions or during daily life later in the rehabilitation journey. Compared with rigid exoskeletons, soft robots have a lower payload. The lower payload can be attributed to the flexible materials used, which allow for greater degrees of freedom and adaptability, but resultingly lack the rigidity required to match the force output of their rigid alternatives. This can pose challenges for use in patients with moderate-to-severe spasticity (Modified Ashworth Scale >2) or in applications requiring higher assistive forces/torques (e.g., supporting the entire weight of the arm).

As soft robotics promise to be simpler and safer than their rigid counterparts, clinicians may be more inclined to consider including them in their rehabilitation sessions. However, it is important to clarify that simple and safe do not necessarily directly translate into a well-accepted device. This highlights the importance of iterative design by evaluating and optimizing device features such as this technology’s effectiveness, affordability, operability, perception, and acceptability.^29^ Additional factors such as weight (both total and worn by the user), whether a device is tethered (portable vs wearable), and ease of configuration to patients should also be considered in the design and selection of devices.^30–32^ However, there may also not always be a single best solution that fits the requirements of all end users. Designs can also be modular and adaptable to fit the best context of use; for example, a device that can be wheelchair mounted does not necessarily have to be as small and as light as something worn directly by the end user.

## Existing Clinical Evidence: What Can We Learn From It?

In the following, we review different representative use cases for wearable soft robotics in rehabilitation and assistance and the results reported in feasibility trials. Specifically, we attempted to underline how softness plays a role in each selected application (e.g., design complexity, type and amount of provided assistance, and reported usability), focusing on rehabilitation and assistance provided at different upper limb segments (Table 1).

**Table 1.** Overview of Available Clinical Evidence on Upper Limb Soft Robotics

Upper limb segment	Soft UL rehabilitation technologies evaluated in end users	Technical characteristics—main features				Clinical evaluations			Main findings on usability
		Soft actuation principle	Weight	Portable/wearable^z^	Availability	Hospital	Home	Type of therapy/assistance	
Hand	Tenoexo,^17^ PEXO^31^	Spring blades w/ Bowden cables	105–148 g^17^ + 492 g backpack (without battery)^17,31^	Wearable	Research project	- Stroke (adults) (*n* = 1),^17^ (pediatrics) (*n* = 1, *n* = 4)^31,53^ - SCI (*n* = 1),^17,34^ *n* = 15)^17,31,52^ - Other^a^ (pediatrics) (*n* = 7)^53^	- TBI (Pediatrics) (*n* = 1)^51^	- Active: task-oriented exercises^17,31,52,53^ -Active: support in ADL^51^	- Functional benefit is dependent on the residual abilities of the end user — more severely impaired patients benefit more from the assistance -Clinically meaningful improvement in ARAT (up to +15 points)^17,52^-Good SUS score (mean = 60.6)^52^
	HERO^45^	Combined linear actuated blades and tendon driven	284 g, fully wireless^54^	Wearable	Research project; Open source; Low cost (>US $160)	- Stroke (*n* = 11, *n* = 5, *n* = 9)^45,47,54^	- Stroke (*n* = 4)^46^ - SCI (*n* = 1^b^)^46^	- Active: task-oriented exercises^45,47,54^-Active: support in ADL^46^	- Three published user-centered iterative design cycles^45,47,54^-Improvements in GAS, BBT, MAL-AOU, MAL-QOM, and FM-UE over a training period - Good SUS score (72–99)
	Ironhand, Carbonhand	Tendon driven	85 g (+600 g control unit)	Wearable	Commercial device (Bioservo)	—	- Self-perceived decline of hand function (*n* = 91^c^),^42^ (*n* = 13),^48^ (*n* = 46)^49^, (*n* = 63)^50^	- Active: support in ADL^42,48–50^	- First published trial of its type (pilot randomized control at home for extended time)-Feasible to use for assistance and therapy at home for an extended period - Good SUS score (mean = 73)^42^-Improvements in hand function was generally maintained 4 weeks later^49^-High grade reported for donning signifying independent usage at home is feasible^48^
	EsoGLOVE^55^	Pneumatic, fabric-reinforced soft actuators	180 g (+1260 g actuator)	Portable	Research project turned company (Roceso Technologies)	- Stroke (*n* = 2, *n* = 9)^55,74^	—	- Active: ROM and strength^55,74^-Passive^74^	- Grasping performance improved with assistance (based on time and task completion metrics)-Good patient feedback on USE questionnaire & custom questionnaire (≥5/7 on Likert scale)
	Exo-Glove,^41^ Exo-Glove Poly II^32^	Tendon driven	194 g,^41^ 104 g (+ 1140 g actuator)^32^	Portable	Research project	- Healthy (*n* = 1)^41^-SCI (*n* = 1 ^d^, *n* = 2)^32,41^	—	- Active: task-oriented training^32,41^	- Grasping performance improved while wearing the glove (based on grasping and lifting force)-Highly adaptive structure and mechanism
	Cappello et al., 2018^30,33,35–37^	Pneumatic, fiber-reinforced actuators	77 g (+5 kg actuator)	Wearable	Research project	- SCI (*n* = 9^e),^30^ (*n* = 3f),^35^ (*n* = 13e^)^37^	- Stroke (*n* = 10)^36^	- Active: task-oriented training^30,33,35–37^-Active: support in ADL^37^-Passive^36^	- Largest improvements in TRI-HFT were found in participants with the lowest baseline scores^30^-Participants shared that they would be willing to use the device all day due to its low weight and minimal obtrusiveness - SUS score shows likeliness to accept a soft wearable glove for daily usage^30^ (77.2 +/- 14.7)^36^-9/10 participants could don/doff and use the device independently^36^
	PneuGlove^38–40^	Pneumatic	Not specified	Wearable	Research project	- Stroke (*n* = 15),^38^ (*n* = 7),^40^ (*n* = 3)^39^ - Healthy (*n* = 3)^39^	—	- Active: task-oriented training^38–40^	- n/a
	Kim et al., 2022^43,44^	Cable driven	Not specified	Portable	Research project	- Stroke (*n* = 8)^44^, (*n* = 5)^43^ - Healthy (*n* = 6),^44^ (*n* = 9)^43^	—	- Active: strength and ROM^44^-Active: task-oriented exercise^43^	- With device assistance, increase of average workspace of the thumb increased by 43% (distal-proximal), 207% (dorsal-palmar), and 248% (radial ulnar) directions -Assistance allows for more stable grasping and manipulation in various grasp patterns -Combining robotic hand with mirror hand therapy resulted in greater mean cortical activation on the motor cortex contralateral to the affected hand
Shoulder/Elbow	O’Neill et al., 2020^58,61^	Pneumatic	150 g (+3600 g actuator)	Wearable	Research project	- ALS (*n* = 10)^58^ - Stroke (*n* = 5)^61^	—	- Active: task-oriented training^58^-Active: strength and ROM^61^	- Good SUS score (79.2+/- 9)-Participants were confident using the robotic wearable - The system’s softness allows it to be highly transparent and allows for natural movement when deflated
	Myoshirt^56^	Tendon driven	520 g	Portable	Research project	- Healthy (*n* = 10) - Bethlem muscular dystrophy (*n* = 1) - SCI (*n* = 1^f^)	—	- Active: task-oriented training^56^	- Functional ROM is unaffected when wearing the MyoShirt - 8/10 participants found the device intuitive
	Xiloyannis et al., 2019^75^	Tendon driven	215 g^59^	Portable	Research project	- Healthy (*n* = 8, *n* = 4)^75,76^ - Stroke (*n* = 10)^59^	—	- Active: task-oriented training^59,75,76^	- Good alignment and fit; tolerable to wear for the whole 90 min session without needing adjustment and showing acceptable skin tolerability^59^-Positive initial acceptance; good subjective ratings relating to impairment reduction, functional assistance, and user experience^59^
	CRUX^62^	Tendon driven	1300 g	Wearable	Research project	- Healthy (*n* = 4) - Stroke (*n* = 1)	—	- Active: task-oriented training^62^	- No significant changes to the user’s natural ROM while wearing
	Lotti et al., 2023^77,78^	Tendon driven	2 kg	Wearable	Research project	- Multiple Sclerosis (*n* = 8)	—	-Active: task-oriented training^77,78^	- The exosuit allowed for an improvement in ROM without hindering the wearer’s motion
	Koh et al., 2017^79^	Pneumatic, fiber-fabric-reinforced actuator	Not specified	Portable	Research project	- Healthy (*n* = 6)	—	- Active: strength and ROM^79^-Passive^79^	- n/a

^a^ 2 TBI, 1 ependymoma, 2 CP unilateral spasticity, 1 rhabdomyolysis, 1 neuropathy.

^b^ ASIA C8 Motor Stage 1.

^c^ For example, rheumatoid arthritis, osteoarthritis, and stroke.

^d^ SCI C6.

^e^ SCI C4–C7.

^f^ SCI C4/5.

^g^ SCI sub C4/5 complete.

^z^ Portable, can be easily transported; Wearable, entire system can be worn and carried while using.

ADL, activities of daily living; ALS, amyotrophic lateral sclerosis; ARAT, action research arm test; BBT, box and block test; CMC, carpometacarpal joint; FM-UE, Fugl–Meyer Upper Extremity Assessment; GAS, Goal Attainment Scale; IP, interphalangeal joint; MCP, metacarpophalangeal joint; MAL, motor activity log (AOU, amount of use, QOM, quality of movement); ROM, range of motion; SUS, System Usability Scale; SCI, spinal cord injury; TRI-HFT, Toronto Rehabilitation Institute–Hand Function Test; TBI, traumatic brain injury; USE, Usefulness, Satisfaction, and Ease of Use Scale.

### Distal upper limb soft robotics—the hand and wrist

With its high number of joints and highly variable size, the hand poses important challenges for rigid assistive structures. As such, various soft hand exoskeletons have emerged in the last decades, taking advantage of softness to help naturally shape hand posture around objects intended to be grasped. Researchers and engineering teams have approached this challenge from different perspectives, such as using pneumatic gloves,^30,33–40^ tendon-based systems mimicking the muscle structure of the hand,^32,41–44^ and hybrid solutions,^17,31,45^ which enhance functionality and versatility. Many of these devices were preliminarily evaluated in pilot clinical studies with pathologies ranging from stroke,^17,36,38–40,43,44,46,47^ spinal cord injuries (SCIs),^17,30,35,37,46^ general upper-extremity impairments,^42,48–50^ and muscular dystrophy.^33^ Many of the listed examples also evaluated the usability of the devices.^17,30,31,42,45–47,51–59^ Users generally received the devices well, praising these examples’ portability, weight, and intuitiveness. Participants also generally saw improvements in their clinical scores (i.e., immediate benefit of wearing the assistive device), showing the functional benefit of using these devices to compensate for a loss of hand motor function.

Several studies^36,42,46,50,51^ have gone beyond testing these devices in controlled settings, instead using them at home to test their efficacy in real-life scenarios and conditions. These studies aimed to validate the devices’ efficacy, usability, reliability, and user-friendliness to provide valuable insights into the practical applications of this technology in uncontrolled settings. For example, the HERO glove was used by stroke and SCI patients (*n* = 5), training first in the clinic for five days, followed by two days of unsupervised use at home during everyday tasks and prescribed exercises.^46^ The usage of PEXO has also been investigated in a single case study with a child at home.^51^ Interestingly, the study highlighted the feasibility of using such a soft exoskeleton at home without specific technical knowledge and after only minimal training (two sessions), graduating from a controlled setting with therapist guidance to uncontrolled usage at home.^51^ Although both devices have shown benefits in terms of independence and recovery, feedback such as improvements in grip strength, ease of donning, robustness, and control is needed to restore the full capabilities of the affected hand, especially in an uncontrolled environment.

### Proximal upper limb soft robotics—the shoulder and elbow

Both the shoulder and elbow joints prove particularly challenging to assist due to the high torque required to offset the weight of the arm. The shoulder has an added layer of complexity due to its high degrees of freedom, whereas most musculoskeletal disorders affecting the elbow also affect contiguous joints such as the shoulder or the hand, thus making it difficult to isolate. In this work, we present shoulder and elbow soft rehabilitation and assistive devices, grouping them together as proximal arm supports.

The primary actuation methods also converge to pneumatics,^58,60,61^ cable-/tendon-driven approaches,^56,62^ or hybrid solutions. These devices were tested in various populations such as healthy,^60,62^ stroke,^59,61^ Bethlem muscular dystrophy,^56^ amyotrophic muscular sclerosis,^58^ SCI,^56^ and general upper-extremity impairments.^62^ Results from these studies ranged from improving range of motion (ROM),^59,61^ reducing muscular effort,^56,59,60^ and increasing muscular endurance.^56^

## Discussion

In this work, we highlight the use of soft robotics for clinical usage in upper limb rehabilitation and assistance. We provide an overview of several use cases of different applications of the technology to underline the feasibility and usability of soft robotics in clinical applications. In these studies, we see from patients, clinicians, and secondary stakeholders the positive impact soft robots can have across different joints of the upper limb. Quantitatively, the amount of assistance upper limb soft robots can provide can be suitable for rehabilitation applications, and when using the technology, patients showed clinically meaningful improvements in their functional abilities. Qualitatively, the feedback received about the devices was positive—praising features inherent to soft robotics, such as simplicity, ease of use, and comfort.

### Use cases and applications of the technology

Softness in upper limb rehabilitation robotics has allowed for the development of more lightweight and portable solutions. This portability eliminates the restrictions of where this technology could be used, thus increasing its potential and applicability. While portability is vital, we see the true potential of these devices in fully wearable solutions. As such, the devices could be used therapeutically or for assistance, irrespective of time or place. In fact, the way soft robotics are being used in therapy can be grouped into four categories as follows: passive exercises, active strengthening and ROM, active task-oriented exercises (in a therapy context), and active support in activities of daily living (ADL) (general assistance outside of therapy). This speaks to the versatility of soft robotics, showing that they can be used in different contexts of use (e.g., in clinic or at home) and use cases (e.g., robotic mirror therapy^43^). While passive exercises can be supported, active exercises are favored as seen in 32 of the 37 reported studies. This could be attributed to the multidirectional support of soft robots, which are able to provide sufficient and adaptable levels of support (necessary for the unpredictability of ADL) all in a small and portable form factor.

The presented use cases show that there is still a gap between portability and wearability, which is particularly apparent depending on the device’s actuation method. Especially when assisting the hand, where smaller forces are required, pneumatic systems which typically require bulkier air canisters may prove less favorable compared with cable or tendon-based systems that can have smaller form factors; however, innovations in miniaturized electric pumps could enhance the portability of future pneumatic systems. Despite the progress in wearability supported by soft structures, it was also evident within the reviewed devices that there is still a lack of evaluation outside of a controlled environment (e.g., during daily life at home). Specifically, there were no instances of any proximal arm device being tested at home. Whether this has to do with the current state of technology, the feasibility of performing a study in such a challenging environment or whether there is even a need for this technology in the home remains debatable. Regardless, researchers should plan appropriate studies or identify new use cases of the technology to help propel the technology readiness level of the devices and thoroughly evaluate their usage in real-world environments to get a complete picture of their potential clinical impact.

The decision is not as clear-cut as choosing between soft or rigid robotics, but researchers and clinicians should focus more on selecting the most appropriate technology (or combination of technologies) for a specific use case. The transition to soft robotics does not make rigid robotics obsolete. Instead, it complements them by covering a broader spectrum of rehabilitation needs, from heavy support in early-stage recovery to subtle assistance in later stages or daily activities as assistive technology. It is also not realistic to evaluate the effect of softness in a system directly, as any direct comparison to a rigid counterpart would face many methodological challenges stemming from the different design approaches. As such, the potential benefit softness can bring in a system remains mostly conceptual and must be seen in terms of how it can ease the implementation and usability in specific use cases. Looking at assisting the hand, for example, by relying on the softness of the mechanism, a hand exoskeleton or a prosthesis can naturally adapt to objects that impose their movement due to the compliance of the device.^17,63^ In this case, softness can simplify the device, making it more intuitive for the end user. Now, considering lower limbs and gait assistance, it is a much more repetitive and predictable movement from the end user and requires a much greater amount of support. In this case, rigid links may better provide users with the precision and force they need to support them during the gait cycle. Recent reviews on soft robotics to assist mobility specifically highlighted the current limitations of soft robots when it comes to providing the larger forces required to support gait, especially at the level of safety, adaptability, ease of use, weight, and cost.^26,64^ Together, rigid robotics and soft exosuits provide a comprehensive range of solutions, ensuring that individuals across different stages of impairment and recovery can receive appropriate support and assistance. By combining the high-precision, structured support of rigid robotics with the adaptable, comfortable assistance of soft exosuits, the field of rehabilitation can cater to a more diverse range of patient needs, promoting better outcomes across the continuum of care.

### Technical applications and outlook

When developing upper limb robotics for rehabilitation and assistance, one should also not limit themselves to a strict definition of softness. In most applications, it is sensible to consider hybrid solutions combining a soft structure with rigid components (e.g., the basic skeleton of the structure) to ensure proper fixation to the users’ limb, for example. Conversely, adding a layer of softness to a previously rigid system (e.g., flexible joints and series elastic actuators) can help to absorb shocks or interact with an unexpected environment.^65^ Irrespective of the application, careful design is essential for adequate force application, safety, and comfort. On the one hand, designers should take advantage of the flexibility provided by soft robotics, the lesser need for correct alignment and the added benefit of easier customization. However, special considerations must be made when managing the upper limb with spasticity, especially in the fingers and thumb. Customization of the appropriate parameters, such as the torque and ROM allowed, must be precise to minimize risk of strain injuries. For example, excessive force application may pull the joints into hyperextension, thus causing injury and pain. On the other hand, one still needs to consider the importance of good design, for example, accounting for correct placement of anchor points in cable-driven systems or alignment of air chambers in pneumatic systems to maximize force output while minimizing any discomfort felt by the end user. Weight and portability (including considering whether the device is tethered) are essential to consider for later acceptance and applicability of the devices in the clinics or at home.^25^

The selection of control methods should also be considered when selecting which solutions are most appropriate for a specific rehabilitation application. The control of soft materials presents intricate challenges due to their nonlinearity and time-varying properties.^66^ Soft robots often leverage bioinspired control strategies, mirroring neural coordination for natural movement. However, integrating sensors and adaptive controls can provide real-time feedback and adjustments to the wearer’s movements, enhancing the rehabilitation process through personalized assistance. Finally, to increase the overall acceptance and effectiveness of soft robotics in rehabilitation and assistance, additional factors such as the careful selection and application of intention detection strategies^67^ should be considered. In this study, besides the most conventionally used solutions relying on buttons or electromyography, other approaches leveraging smart materials or pressure sensors could be integrated into the soft structures.^68^

Combining different rehabilitation approaches with soft robotics should be further investigated. For example, a promising approach could be to combine functional electrical stimulation with soft robotics, which can help to either provide more muscular strength or help ease spasticity experienced after stroke, all while the soft structures provide the guidance needed to perform the desired movement.^69,70^ These two independently would assist the patient, but combined, they may bring even more usage and benefit.

Finally, it is essential to consider the cost and feasibility of scaling rehabilitation devices, even from the earliest stages of product development. Regardless of the funding source (self-funded, covered by insurance, included in public health care, etc.), cost will always remain an obstacle in the eventual acquisition and acceptance of assistive technologies. From a commercial perspective, scalability and manufacturability should be evaluated throughout the design stages. Especially in soft robotics, looking at textiles or molded elastomers, it can be challenging to find reputable manufacturers who have the relevant experience needed to manufacture these components or new manufacturing skill sets need to be developed to bring soft devices past the research prototype stage. These obstacles will inevitably make it difficult to scale the technology and will thus drive up costs compared with more conventional rigid robotics. One possible approach is to create open-source solutions (e.g., the HERO glove)^47^ that can, for example, be manufactured using rapid prototyping and hand tools to make technology more widely accessible. While 50 HERO Gloves have been produced, in-person training, quality assurance, and financial compensation have been provided in all known build cases. While this is a creative way to increase the accessibility of the technology and foster community involvement in the design process, it comes with challenges, such as the need for quality assurance to ensure devices with intricate soft robotic components function as intended and are safe to use, funding models that reimburse robotic components, and data management systems that monitor device usage and collect user feedback on device performance and future needs.

### Clinical validation

Our overview of clinical study evaluations of upper limb soft rehabilitation technologies (see Table 1) highlighted that there are still limited devices that end up being clinically validated with end users. While most devices show feasibility, little is reported on the clinical benefits, usability is often only partially evaluated, and most studies are short pilot studies.

As the field continues to generate new technical developments, rather than continuing to publish primarily technical articles, they should be supplemented with more preliminary user testing and usability evaluations. The direct interaction with groups of users can help better identify early limitations and the potential for technological improvements and drive the necessary fast design iterations, considering the feedback from multiple stakeholders. This collected feedback can also help to understand how end users and clinicians perceive the technology. From the review of existing clinical studies (Table 1), it became evident that usability evaluations are underreported. This could be attributed to a lack of standards and experience in performing these types of studies. Usability evaluations should include both standardized and customized tools to help establish usability benchmarks with comparable devices. Resources such as the Usability Toolbox^71^ can be used to help find appropriate usability measures depending on specific contexts of use.

Especially in the early stages of technological development, frequent small-scale tests may prove more valuable than resource-demanding randomized-controlled trials (RCTs).^72^ RCTs rely on high samples, standardized interventions, and homogeneous populations in the tested groups. All of these are hardly compatible in the field of wearable robotics for rehabilitation, as patients’ impairment levels and rehabilitation goals may widely vary and with the growing evidence calling for more personalized rehabilitative interventions (e.g., in terms of tailoring hardware, but also of the specifics of the intervention and its duration and intensity, factors that are hardly controllable outside of clinical settings).^73^ While RCTs remain necessary, one should still better value feasibility studies in smaller groups of end users, where the potential of the technology, its safety, and usability can be more closely evaluated together with all involved stakeholders.^73^ In recent years, teams of engineers and therapists in hand rehabilitation have successfully adopted this user-centered design approach (e.g., tenoexo, Carbonhand, HERO glove, EsoGLOVE Pro, Exo-Glove), leading to some accessible solutions through retail and open-source manufacture.^42,47,55^ Such studies have typically recruited less than 10 participants per group and have 1–7 use sessions. This enables case-by-case analysis of the barriers and facilitators to adoption and generates design guidance for future iterations before large investments in sizable clinical trials. Within these smaller scale studies, it is still imperative to have a heterogenous population to ensure that it is well understood how the technology affects the largest representation of individuals.

It should be noted as a limitation of this perspective article that the included literature was not collected systematically. While the listed work was compiled with the help of international experts in the field and with the goal of providing a general overview of the current state of clinical evidence of soft robotics in neurorehabilitation and assistance, there could be a selection bias, and the list of available literature may not be completely exhaustive.

### Recommendations

To conclude this perspective article, we derive from our analyses a set of five important recommendations aimed at developers and end users of soft robots for upper limb rehabilitation and assistance. With these, we attempt to highlight some necessary directions to consider further and report the benefits of soft robotics in rehabilitation and, second, to help make these systems more accessible to patients who would benefit from these.

1. **Technical development:** Work to transform soft robotics from portable to wearable to maximize their impact and applicability in daily life at home. This would open the door to combining therapy and assistance, potentially impacting rehabilitation dose and clinical outcomes.
2. **Design of clinical validations:** Dedicate more time and resources to evaluate and modify existing devices rather than developing new ones that will remain at the prototype stage.
3. **Usability evaluations:** Perform and report well-rounded usability evaluations with end users, clinicians, and other involved stakeholders. Use a combination of usability measures (e.g., mixed-method usability evaluation) with standardized scales to allow for benchmarking devices and clinical protocols.
4. **Reporting on the contributions of softness:** Report on the specific contributions of softness in designs to better identify the benefits and gaps in which this new category of rehabilitation devices excels. More effort should be put into valorizing the unique benefits of softness in specific use cases.
5. **Accessibility of technology:** Consider the future scalability and accessibility of the technology to maximize its outreach.
